# An Occupational Heat–Health Warning System for Europe: The HEAT-SHIELD Platform

**DOI:** 10.3390/ijerph16162890

**Published:** 2019-08-13

**Authors:** Marco Morabito, Alessandro Messeri, Pascal Noti, Ana Casanueva, Alfonso Crisci, Sven Kotlarski, Simone Orlandini, Cornelia Schwierz, Christoph Spirig, Boris R.M. Kingma, Andreas D. Flouris, Lars Nybo

**Affiliations:** 1 Institute of BioEconomy—National Research Council, 50019 Florence, Italy; 2 Centre of Bioclimatology—University of Florence, 50144 Florence, Italy; 3 Federal Office of Meteorology and Climatology, MeteoSwiss, Zurich Airport, 8058 Zurich, Switzerland; 4 Meteorology Group, Dept. Applied Mathematics and Computer Sciences, University of Cantabria, 39005 Santander, Spain; 5 Department of Agricultural, Food, Environmental and Forestry Sciences and Technologies, University of Florence, 50144 Florence, Italy; 6 Department of Nutrition, Exercise and Sports, University of Copenhagen, 2100 Copenhagen, Denmark; 7 Unit Defense, Safety and Security, The Netherlands Organisation of Applied Scientific Research, 3769DE Soesterberg, The Netherlands; 8 FAME Laboratory, Department of Exercise Science, University of Thessaly, 42100 Karies, Greece

**Keywords:** worker, customized forecast, Wet-Bulb Globe Temperature (WBGT), risk level, heat adaptation, work breaks, hydration, European Center for Medium Range Weather Forecasts (ECMWF), global warming

## Abstract

Existing heat–health warning systems focus on warning vulnerable groups in order to reduce mortality. However, human health and performance are affected at much lower environmental heat strain levels than those directly associated with higher mortality. Moreover, workers are at elevated health risks when exposed to prolonged heat. This study describes the multilingual “HEAT-SHIELD occupational warning system” platform (https://heatshield.zonalab.it/) operating for Europe and developed within the framework of the HEAT-SHIELD project. This system is based on probabilistic medium-range forecasts calibrated on approximately 1800 meteorological stations in Europe and provides the ensemble forecast of the daily maximum heat stress. The platform provides a non-customized output represented by a map showing the weekly maximum probability of exceeding a specific heat stress condition, for each of the four upcoming weeks. Customized output allows the forecast of the personalized local heat-stress-risk based on workers’ physical, clothing and behavioral characteristics and the work environment (outdoors in the sun or shade), also taking into account heat acclimatization. Personal daily heat stress risk levels and behavioral suggestions (hydration and work breaks recommended) to be taken into consideration in the short term (5 days) are provided together with long-term heat risk forecasts (up to 46 days), all which are useful for planning work activities. The HEAT-SHIELD platform provides adaptation strategies for “managing” the impact of global warming.

## 1. Introduction

Meteorological observations worldwide reveal significant increases of heat-stress conditions and future climatological scenarios report that we should expect far worse situations even in the most optimistic projections [1,2]. Workers, and above all outdoor manual workers, represent an important part of the population potentially vulnerable to heat stress [3,4,5]. In particular, work that involves high levels of physical exertion—such as heavy lifting and manual labor carried out for example by farmers, construction workers, fire-fighters, miners, soldiers, and manufacturing workers operating near artificial heat sources—are particularly affected since individuals tire faster and metabolize heat less effectively under exertion [3,4,6]. Workers are often exposed for many hours to direct solar radiation or artificial radiant heat, and in several cases wearing personal protective clothing and equipment that significantly aggravate heat stress by limiting body heat loss. An advanced working age or the potential interaction between heat and chemical substances (i.e., pesticides and fertilizers), used i.e., in agricultural activities, represent other important heat-related vulnerability factors. For economic reasons, workers may need to work during hot weather conditions, which impose an occupational heat stress; it is a public health issue. However, appropriate adaptation strategies could help to avoid heat-related health problems, and also limit the typical productivity loss that occurs during the warmer period of the year [7,8]. Although workers contribute enormously to economic growth, they are often overlooked in discussions about the effects of heat [9,10], and specific heat warning systems for occupational purposes are actually unavailable internationally.

An accurate and timely heat–health warning system (HHWS) represents one of the core elements nested in a wider Heat–Health Action Plan, which encompasses and directs all preventive measures to be taken to protect the population from the effects of environmental heat exposure [9,11]. At present, HHWSs aim at protecting the general population or people considered most vulnerable, such as the elderly population [12,13], even if in recent studies [10,14] young workers were found to be as vulnerable and at increasing risk of occupational injuries with high temperatures. Various metrics to define the effects of heat on health have been developed [9,15,16], based on different thermal (i.e., single-metric based on air temperature or heat stress index; heat budget models; air mass-based synoptic climatological approaches) and health indicators (generally mortality data, but also morbidity indicators could be used if available). In this way, different heat threshold levels based on epidemiological (i.e., city-specific heat-related mortality thresholds) or climatological (i.e., specific percentiles of the local distribution of minimum and maximum temperatures) evidences, or even based on heat stress levels assessed through specific thermal stress indices, have been developed and are currently used to issue heat warnings in HHWSs [9,17]. Concerning the occupational sector, a HHWS should be more focused on heat stress than mortality events or other health indicators addressed to the general population. This is because heat stress for workers represents a public health concern [5] and worker health and performance are affected at much lower environmental heat strain levels than those directly associated with higher mortality. When working in the heat, skin blood flow and sweat rate increase to allow for heat dissipation to the surrounding environment (thermoregulatory adjustments), thus increasing risk of heat-related injuries, kidney diseases and generally, physiological strain leading to dehydration [18,19]. A recent study [20] revealed that about 70% of workers initiate work with a suboptimal hydration status, meaning that workers are dehydrated at onset of work and that rehydration from day to day may be a bigger issue than failure to drink during the working shift. The higher heart rate associated with dehydration signifies an overall elevation of cardiovascular strain [21]. Dehydration may have an even larger impact on performance in cognitive occupational settings where people are exposed for prolonged periods to high heat stress levels and fail to prevent hypo-hydration [20]. Surely, a good adaptation to heat can help to prevent many negative consequences, even if it has recently been highlighted that heat acclimation may not be sufficient to protect against hyperthermia when complex tasks are performed [22]. Thus, the availability of timely heat stress warnings calibrated on specific work activities and accounting for the clothing worn would make it possible to reduce heat-related performance losses, especially in the case of prolonged heat exposure. 

Due to the different needs of the occupational sector compared to the general population, a HHWS for occupational purposes should have some main characteristics. In particular:

- It should be personalized; that is, based on the physical demands of the job as well as on workers’ physical, clothing, and behavioral characteristics and on the work environment;
- it should include short-term suggestions useful to help heat adaptation for workers;
- it should contain long-term heat risk information for planning/organizing work, which is useful for employers, organizations, and operators in charge of safeguarding health and productivity in various occupational areas.

To achieve this goal, a meteorological forecasting model for different lead times (with forecasts up to about one month) is necessary, as well as the use of a thermal stress indicator able to provide detailed information in various situations. Then, the metric used should account for the level of physical activity performed by a worker, which will obviously be influenced by personal physical characteristics (e.g., weight and height), the clothing worn, and the working environment, differentiating between a worker exposed to solar radiation, or other heat sources, and a person working in the shade.

At the international level, there is presently no example of a HHWS specifically targeting workers and capable of meeting the main characteristics listed above. At European level, an important contribution on this topic has been provided by the European project “Integrated inter-sector framework to increase the thermal resilience of European workers in the context of global warming” (HEAT-SHIELD) [23] that aims to develop solutions to protect the health and productivity in workplaces from excessive heat in the context of climate change. In 2017, a first prototype of an occupational HHWS for entire Europe for a timeframe of four upcoming weeks was developed within the frame of HEAT-SHIELD. In 2018, an online open access service (website platform) was officially launched to help industry and society anticipate threats by heat stress to workers’ health and productivity.

The aim of this paper is to present and describe the characteristics of the website platform “HEAT-SHIELD occupational warning system” [24], currently operating for the entire Europe and representing one of the main outcomes of HEAT-SHIELD. 

## 2. Materials and Methods 

In this section, descriptions of the weather forecast model and the heat stress indicator used to develop the website platform are provided, together with the main outputs of the HEAT-SHIELD platform and a forecast verification analysis.

### 2.1. The Weather Forecast Model

The HEAT-SHIELD platform was developed on the basis of the monthly ensemble forecasts of the European Center for Medium Range Weather Forecasts (ECMWF) [25]. The forecast ensemble consists of 51 members, with the control being the member with initial conditions corresponding to the best estimate of the operational analysis. All members (and the control simulation) represent equally probable atmospheric situations. This operational forecast model provides monthly numerical weather forecasts twice a week, initialized on Monday and Thursday at 0 UTC, and are referred to as ECMWF extended range predictions (ENS-EXT). The current ENS-EXT system has a horizontal resolution of 0.2° × 0.2° (lat × lon, ~18 km mesh size) for the first 15 days and 0.4° × 0.4° (~36 km) from days 16 to 46. Daily forecast values of temperature, humidity, radiation, and wind speed are bilinearly interpolated onto the desired coordinates. 

**Figure 1:**
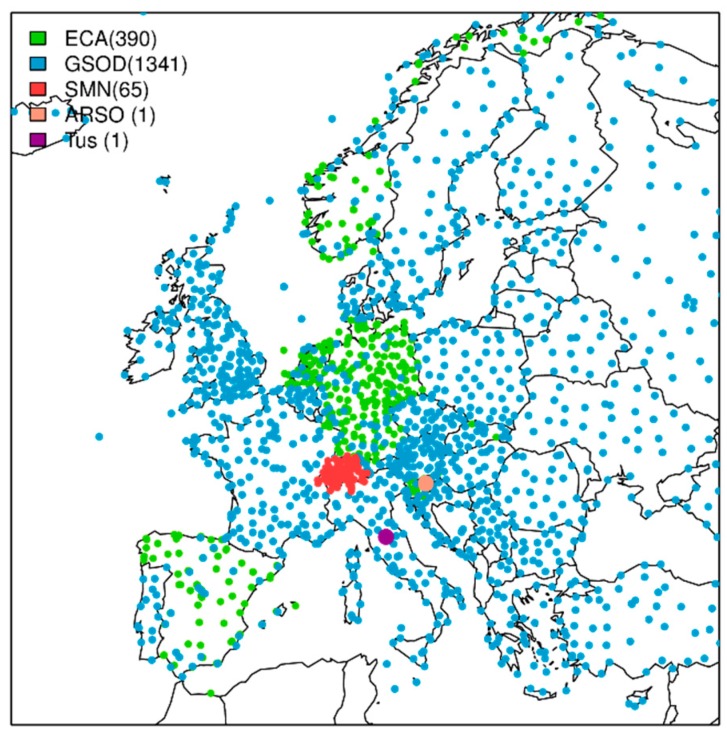
European stations which comprise the observational dataset of the HEAT-SHIELD platform, from ECA & D (green), GSOD (blue), SMN (red), ARSO (pink) and Tuscany (purple).

Due to the coarse spatial resolution and systematic model biases of the ensemble forecast model, it is certainly not suitable for providing detailed information related to the heat warning for workers. Location-specific forecasts rather than gridded forecast products were developed through downscaling and bias correction procedures, namely empirical quantile mapping (hereafter EQM) [26]. This method establishes a quantile-dependent correction function between the observed and simulated distributions [27,28,29]. EQM was first calibrated with paired forecasts and observations from the past 20 years in a lead-time dependent manner [30]. Secondly, the EQM corrections were applied to the actual forecasts. The correction procedure was performed separately to each of the input variables of the heat stress index. In this way, location-specific forecasts were provided for sites where sufficiently long observation records (20 years) of the relevant meteorological variables exist. For this reason, several meteorological datasets were combined in order to get a representative and dense enough, ground-based observational dataset over the entire Europe (Figure 1):

1. The European Climate Assessment and Dataset project (ECA & D) [31] was the primary source for the observational dataset. There is, however, a limited number of stations with non-standard parameters such as humidity and wind (see green points in Figure 1). Thus, the following datasets were used to complete the full set of stations.
2. Dataset of the Global Surface Summary of the Day (GSOD, see blue points in Figure 1) from the National Oceanic Atmospheric Administration (NOAA). Stations exceeding more than 20% of missing values in ECA & D and GSOD in the period between 1996 and 2016 were removed from the dataset.
3. The Swiss national observing system SwissMetNet (SMN, see red points in Figure 1) [32].
4. A couple of stations from HEAT-SHIELD case studies were included in the set of stations: Data from one station in Celje (Slovenia), near the Odelo d.o.o. manufacturing plant [33], provided by the Slovenian Environment Agency (ARSO), and from one station in Arezzo (Tuscany, Italy) provided by Regional Service of Tuscany (CFR).

The resulting observational dataset contains daily measurements of air temperature, dew point temperature, and wind speed from 1798 meteorological stations across Europe and covering a period of 20 years (from 1998 to 2017 for summer 2018 forecasts). As only few of these stations also provide radiation measurements, satellite data for the same period as the other variables were used as best estimates for radiation observations. Specifically, the surface incoming solar radiation product from EUMETSAT’s Satellite Application Facility on Climate Monitoring (CM SAF) [34] was used. The reader is referred to Casanueva et al. [2] for more details on the observational datasets.

### 2.2. Heat Stress Indicator

Based on the target group of the HEAT-SHIELD platform, i.e., workers engaged in outdoor activities exposed in the sun and in the shade, the Wet-Bulb Globe Temperature (WBGT) index was used as the primary heat strain indicator because it can be calculated (estimated) from standard weather and climate model data as well as measured locally at workplaces.

WBGT was originally developed by U.S. military ergonomists in the 1950s [35] and is currently widely used and internationally recognized [19,36] as a method for assessing heat stress conditions specifically in military [37], occupational and sports fields [38,39,40]. WBGT (unit = °C) considers the combination of the natural wet bulb temperature (dependent on humidity, air temperature and wind speed), the black globe temperature (dependent on radiation and wind speed) and the air temperature for estimating heat-stress in the sun (WBGT_sun_, in conditions of direct short-wave radiation) and in the shade (WBGT_shade_, no direct short-wave radiation). The natural wet bulb temperature simulates the cooling of the body via sweat evaporation, while the black globe temperature simulates the heat absorption from short- and long-wave radiations (from the sun, the soil or artificial heat sources in the workplace). These two variables are influenced by both the air temperature and the wind speed. For example, low wind speeds considerably affect black globe temperatures and significant higher values occur with no wind. WBGT shows a large dependence on wind speed when the wind is low and only minor WBGT increases occur above 1 m/s [41].

Based on reference WBGT values, recommendations in term of work–rest cycles and water intake depending on specific work activities are provided by several international organizations. In particular, the International Organization for Standardization (ISO) [36] uses WBGT thresholds to recommend work–rest limits for workers involved in different physical activities and wearing specific clothing in hot environments, in order to avoid a core body temperature exceeding 38 °C [41]. The core body temperature of all humans is maintained close to 37 °C. While some increase in the core temperature beyond this latter threshold may be acceptable, an increase above 39 °C creates health risks [42] that vary from person to person. These variations depend on ethnic group, age, gender, duration of heat exposure and degree of acclimatization; in this way also generating geographical variations [41,43].

At this stage, WBGT is considered to fulfill the purpose for individualized heat warnings, with customized limits for different workers potentially useful for managing policies against the heat effects.

Following the recommendations for calculating workplace WBGT from meteorological data provided by Lemke and Kjellstrom [41], we applied the WBGT implementations of Bernard and Pourmoghani [44] and Liljegren et al. [45] for computing WBGT in the shade and in the sun, respectively. These implementations allow the calculation of both the natural wet bulb temperature, that is the largest component (70%) of WBGT, and the black globe temperature (it contributes 20–30% of WBGT) as required by the WBGT_sun_ and WBGT_shade_ formulas starting from well-established meteorological variables (air temperature, humidity, wind speed, and solar radiation) provided by the weather forecast model. 

The ECMWF model outputs used for the daily WBGT predictions are daily maximum temperature, daily average dew point temperature, and daily average near-surface wind speed. As daily maximum global radiation is not available as a forecast output, we derived this parameter from quantile mapping of daily mean radiation against the observed daily maximum radiation as part of the bias correction. Using these bias-corrected daily values of maximum temperature and radiation, and average humidity and wind, daily maximum WBGT_sun_ and WBGT_shade_ were computed by using the R package HeatStress [46], in this way providing the forecast of the maximum heat stress for each specific day. 

### 2.3. HEAT-SHIELD Platform Outputs

#### 2.3.1. Non-Customized HEAT-SHIELD Platform Outputs

The primary forecast outputs are daily ensembles of the maximum WBGT (both in the sun and in the shade) for each of the 1798 locations. From these WBGT ensembles, probabilities of exceeding any WBGT threshold of interest can be computed, thus allowing to address specific user needs on particular WBGT thresholds. As forecast uncertainty increases with lead time, it is appropriate to use aggregated quantities when attempting to do longer term predictions. Averaging in time or space is a basic way to focus on the more predictable larger-scale components of the atmosphere and allows for extending skill to longer lead times, albeit at coarser (spatial/temporal) resolution [47]. This can be accounted for by creating forecast products displaying the predictions aggregated over several days (aggregated over seven days, from Monday to Sunday) rather than daily information. 

In particular, for each of the four upcoming weeks, a map showing the maximum daily probability of exceeding a specific heat stress condition (WBGT above a specific threshold) scheduled for the week is provided. With the aim to provide an overall picture over Europe, a single WBGT threshold was chosen to assess the heat stress situation potentially detrimental in several outdoor occupational sectors across the continent. The choice was made by using one of the WBGT thresholds described by the ISO standard [36] for which it is required to increase the work breaks with the aim of avoiding that the core body temperature exceeds 38 °C. Trying not to be too alarmist, and taking into account the experience of the HEAT-SHIELD partners and user feedback, a threshold of 27 °C for WBGT_sun_ was exemplary chosen for the weekly operational forecast. For WBGT values above this threshold, the ISO 7243 [36] recommends increasing work breaks for workers engaged in jobs that require a very high or high physical effort depending on whether the workers are acclimatized or not to heat respectively. This information is, however, very general and should be used above all to motivate the user to register on the HEAT-SHIELD platform to obtain customized information based on individual characteristics.

#### 2.3.2. Customized HEAT-SHIELD Platform Outputs

User customized outputs are derived by the personalization of the metabolic rate used for the calculation of the customized recommended alert and exposure limits (RALs and RELs for unacclimatized and acclimatized workers, respectively) as well as correcting the WBGT forecast for insulating effects from clothing and personal protective equipment (PPE) worn by workers. The final output is the customized daily heat risk level forecasted for a specific day.

In detail, the personalized metabolic rate (MR) is assessed on the basis of the body surface area (BSA) and the activity level (ISOlevel). BSA is calculated knowing both the user’s height and the weight (Equation (1)) [48]

(1) $$BSA\;\left(m^{2}\right)=weight\;{\left(kg\right)}^{0.425}\times height\;{\left(m\right)}^{0.725}\times 0.20247$$

Other methods for calculating BSA based on more recent formulas are also available [49,50]. However, the Du Bois and Du Bois formula [48] still represents a standard method widely applied in most medical cases [50,51] and several studies have revealed its strikingly high accuracy [52,53,54].

The ISOlevel is a number measured on a scale from 1 to 5 indicating the user’s activity level (1 being resting, 5 very high metabolic rate) based on the classification of levels of metabolic rate according to the kind of activity from ISO 8996 [55]. 

The personalized MR was calculated based on the Equation (2) that represents a slight modification of the reference table of classification of metabolic rate by category as reported in the ISO 8996 [55].

(2) MR (W)=BSA×ISOlevel×50

The MR assessed is then used to calculate the customized RALs (Equation (3) for unacclimatized workers) and RELs (Equation (4) for acclimatized workers) according to the criteria for a recommended standard of the National Institute for Occupational Safety and Health (NIOSH) [19].

(3) $$RAL\;{(}^{\circ }C-WBGT)=59.9-14.1\;log_{10}\;MR$$

(4) $$REL\;{(}^{\circ }C-WBGT)=56.7-11.5\;log_{10}\;MR$$

Both RAL and REL were developed to protect most healthy workers exposed to environmental and metabolic heat from developing adverse heat-related health effects (i.e., maintain thermal equilibrium): Workers exposed to environmental and metabolic heat below the appropriate NIOSH RALs or RELs will be protected from developing adverse health effects. Healthy workers are those physically and medically fit for the level of activity required by their jobs and wearing the conventional one-layer work clothing ensemble consisting of not more than long-sleeved work shirts and trousers (or equivalent) [19]. When the clothing worn differs substantially from the conventional one-layer work clothing (i.e., more than one layer and/or greater air and vapor impermeability), the rate and amount of heat exchange between the skin and the ambient air will be significantly altered by convection, conduction, radiation, and sweat evaporation. Therefore, the forecasted WBGT is corrected to an effective WBGT (WBGT_eff_) by adding the Clothing Adjustment Value (CAV) as described in ISO 7243 [36].

The final output is the customized heat-related occupational risk level forecasted for a specific day obtained from the percentage ratio between the WBGT_eff_ and the customized RAL or REL (Equation (5)).

(5) $$Risk\;level\;\left(\%\right)=\frac{WBGT_{eff}}{RAL\;\left(or\;REL\right)}\times 100$$

The HEAT-SHIELD platform risk levels are described in Table 1. 

Any user who is initially registered on the HEAT-SHIELD platform is considered an “unacclimatized worker” to heat stress conditions and, for this reason, the RAL is initially used in the calculation of the customized heat risk level. However, after 5 days with at least moderate heat stress risk level (Table 1) forecasted in the short term (the first 5 days of forecasting the heat stress risk of the HEAT-SHIELD platform) during the warm season, the worker is considered “acclimatized” and the REL (higher WBGT limits than RAL) is used for the heat stress risk level calculation. Based on some stakeholder meetings organized as part of the HEAT-SHIELD project, one of the main feedback obtained was that workers are often not able to define whether they are adapted to heat. For this reason, we decided to adopt a simple empirical approach which, based on the available scientific literature, allowed us to consider when a worker can be considered acclimatized to heat. According to previous studies [56,57,58], about 1 to 2 weeks of daily heat exposure are needed to gain adaptation that reduces physiological strain and helps to improve physical work capabilities under a hot environment. Other studies have shown that about 75% of the physiologic adjustments occur within the first 4–6 days of heat exposure [59,60] and a recent study [61] revealed that 5 days of exposure to heat sessions were enough to acclimatize to heat workers involved in hot-climate countries. The 5-day threshold with critical heat stress conditions (in our case with at least a moderate risk level) was therefore used to define when a worker can be considered acclimatized to heat within a warm season.

Recommendations for intra-hourly work breaks and water consumption (hydration) at different metabolic rates described in Table 1 were developed based on the available knowledge from the scientific literature and on the indications reported by the American Conference of Governmental Industrial Hygienists [58], the NIOSH [19], and the ISO 7243 [36].

Further details on the heat stress risk-level-based recommendations are also available:

- Not significant: No special precautions are required and no further breaks than usual are needed.
- Low: You should be able to maintain normal activities. You may experience heat strain (generally low) and increased sweating. Consider clothing adjustment and drink more than normal.
- Moderate: Your water needs will be high. Increase the number of breaks (include small breaks with cooling) and drink frequently. Remember to rehydrate after work/exercise: Be aware that thirst is usually not a sufficient indicator when sweating is high. If this risk level is forecasted during the first summer days, pay extra attention to increase drinking and keep a good hydration status (drink/rehydrate with your meals) outside working hours. Consider adjusting the timing of activities (heavy physical tasks) to the cooler period of the day.
- High: This level is associated with severe heat stress. It is strongly suggested to adjust work—use active cooling, schedule frequent breaks in shadowed or cool areas where you can hydrate. Additional drinking is required (water needs may be more than 1 L/h). If possible, after consulting your doctor, add mineral salts to your meals. Consider adjusting the timing of activities (moderate–heavy physical tasks) to the cooler period of the day.

#### 2.3.3. Forecast Verification 

With the aim to monitor and improve the forecast quality (the ability of a model to correctly predict an event, that is the degree of agreement between the forecasts and the corresponding observations), the forecasts during summer 2018 were verified against observations. For this purpose, a thorough comparison of the daily WBGT forecasts against the corresponding observed values at the 1798 representative meteorological stations used for downscaling and bias correction procedures was carried out. The probabilistic component of ensemble forecasts requires diverse metrics to characterize their quality in terms of accuracy, reliability association, and discrimination [30,62]. In this work, the continuous ranked probability score (CRPS) was used to assess the accuracy of the forecasts [63]. This metric is widely used in forecast verification and represents the ensemble version of the mean absolute error. It is sensitive to the bias in the ensemble mean and to the over- or under-dispersion of the ensemble (i.e., it also penalizes ensembles with large spread even when having a good ensemble mean prediction). The score can be expressed as a skill score (SS) relative to a reference forecast (CRPSS). A positive CRPSS indicates a better performance of the forecast compared to the reference (perfect score of 1), CRPSS = 0 means that the forecast is as good as the reference and negative scores indicate lower skill than the reference. In this work, the CRPSS of the bias-corrected forecasts is obtained considering two possible reference datasets: (1) Raw forecasts (non-bias-corrected) and (2) observations from the past 20 years (which mimic a 20-member ensemble, hereafter climatological forecasts). The former shows potential added value of the bias-corrected forecasts with respect to the uncorrected forecasts, whereas the latter represents the skill of the forecasts with respect to a naive forecast based on climatological observations. Daily CRPS values were averaged into weekly values (week 1 spans from day 5 to day 11, week 2 from day 12 to day 18, week 3 from day 19 to day 25, week 4 from day 26 to day 32). Final CRPSSs were obtained from the weekly CRPS, for each European location. The verification was conducted considering forecasts which span from April to September 2018, i.e., 40 forecasts.

## 3. Results

**Figure 2:**
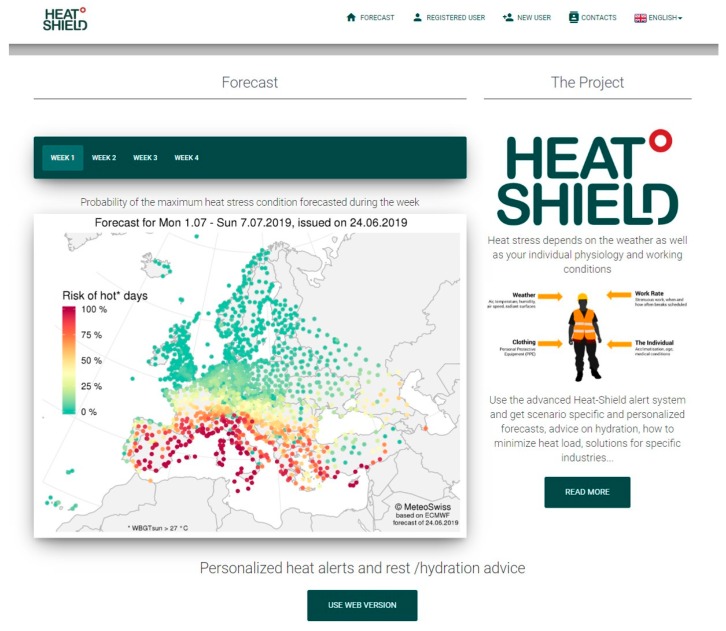
Home page of the HEAT-SHIELD occupational warning system [24].

Besides the general information on the HEAT-SHIELD project, the home page of the multilingual website platform “HEAT-SHIELD occupational warning system” (Figure 2) [24] contains the non-customized heat stress forecasts and a link “use web version” to access the registration to get customized forecasts.

### 3.1. HEAT-SHIELD Platform Interface and Outputs

The map of the weekly maximum probability of exceeding the daily WBGT_sun_ threshold of 27 °C, available for each of the four weeks, is the non-customized heat stress forecast output and is generalized information accessible to everyone without any registration. This information is provided for all 1798 locations (represented by points) for which the forecast is available at European level and is shown by a point-related chromatic scale varying between green (the lowest probability of occurring) and dark red (the highest probability) (Figure 2). Figure 2 shows a clear gradient with high probabilities of WBGT_sun_ > 27 °C in southern Europe, medium probabilities in several central European countries and very low probabilities in the North. This spatial distribution results from the typical climatological conditions of air temperature, with a south-north (latitudinal) gradient.

**Figure 3:**
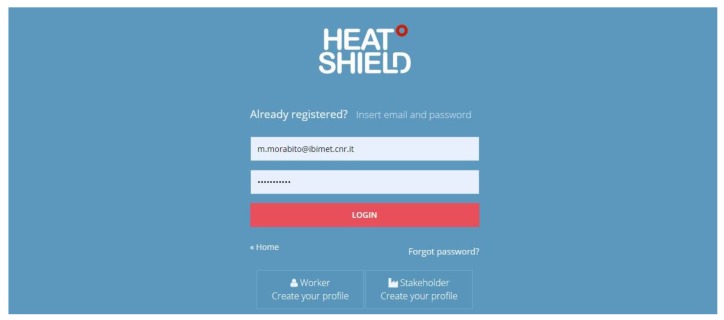
Registration page to access the personalized HEAT-SHIELD occupational warning system outputs.

To access the personalized forecast heat stress alert system, including the suggested rest/hydration advices, a registration is required (Figure 3) by clicking on “USE WEB VERSION”. 

**Figure 4:**
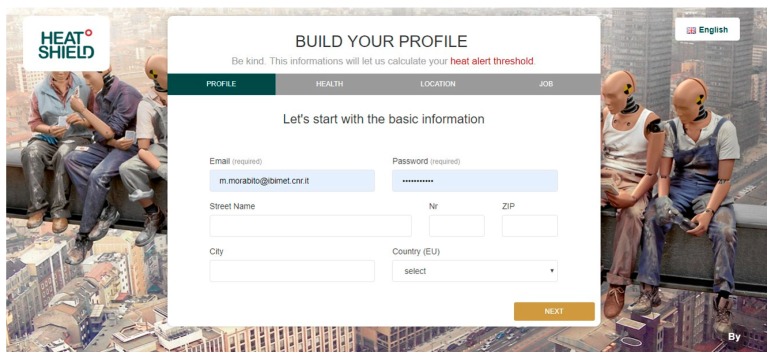
Registration page to create your own profile of personalized heat stress warnings.

The registration process consists, in a first step, of providing an e-mail address (Figure 4), necessary to receive an alert message automatically in the event of a moderate (or high) heat risk level in one of the first 5-day forecasts (the first 5 days of forecasting the heat stress risk of the HEAT-SHIELD platform), and a password to access the user profile at any time and change it if necessary. Subsequently, the user must provide a series of information including the most important for the purposes of calculating the customized heat stress:

- Height (cm) and weight (kg);
- The location, which must be chosen by the user after indicating the exact address for which the forecast is need and double clicking on the available shield (one of the 1798 stations) nearest and with similar altitude to the location of interest;
- The physical activity level (low, moderate, high, and very high);
- The work environment (outdoors in the sun or shade);
- The type of clothing or PPE worn during work.

The registration process can be done by a user identified as a worker or a stakeholder (i.e., employer, competent doctor, or other operators in charge of safeguarding workers’ health). In the latter case, the difference consists in the possibility to select a “standard” worker in terms of height and weight. 

**Figure 5:**
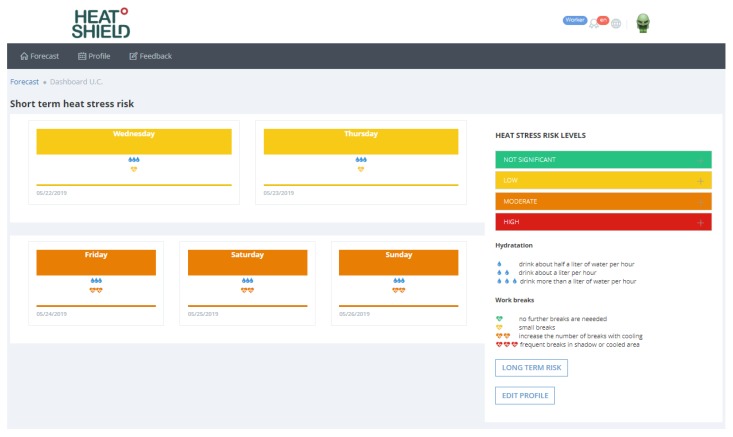
Worker’s heat stress risk and behavioral suggestions to be taken in the short term (the first 5 days of forecasting the heat stress risk of the HEAT-SHIELD platform) available in the own profile of the personalized heat stress warning.

Once the registration is completed, the user can access his/her personal forecast page containing the forecasts of the heat stress risk and behavioral suggestions (in relation to hydration and work breaks recommended) to be taken in the short term, that is for the first 5 days (Figure 5). 

**Figure 6:**
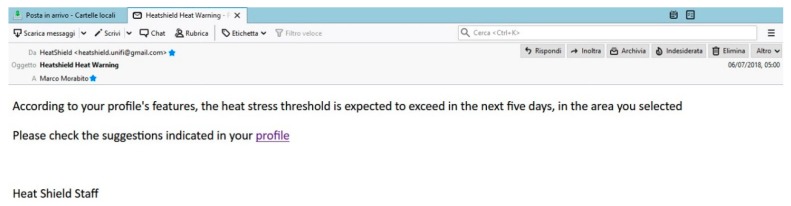
The HEAT-SHIELD warning sent to the user’s e-mail when at least one day with a moderate (or high) heat stress risk level is expected in the short term (the first 5-day forecasts of the HEAT-SHIELD platform).

The short-term warning forecast is updated daily. If at least one day with a moderate (or high) heat stress risk level is expected in the short term (within the first 5-day forecasts), a warning message is automatically sent to the e-mail address provided by the user during the registration process (Figure 6).

**Figure 7:**
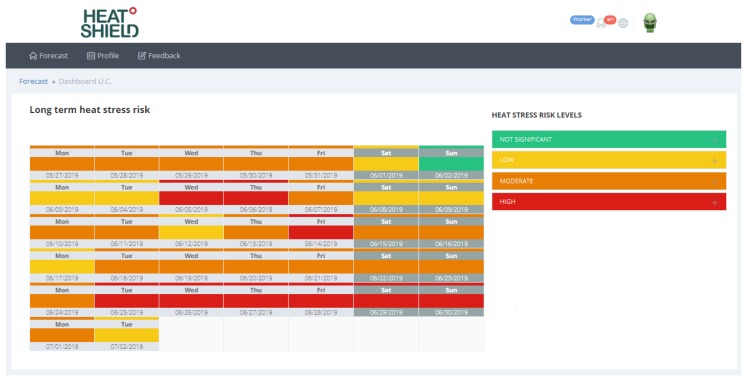
Worker’s heat stress risk in the long term (from the 6th to the 46th day forecasted) available in the own profile of the personalized heat stress warning.

In addition, the worker’s heat stress risk is also available in the long term (by clicking on “LONG TERM RISK”) by means of a colored calendar (Figure 7), from the 6th to the 46th day, updated twice a week, on Tuesday and Friday. In this case, the information is mainly aimed at providing useful information for planning work activities in the long term.

It is also possible to modify the user’s profile by clicking on “EDIT PROFILE” (e.g., changing the physical characteristics, the work effort, the workplace condition, or the clothing worn) and immediately obtaining new short- and long-term heat stress risk forecasts based on the new input data. 

**Figure 8:**
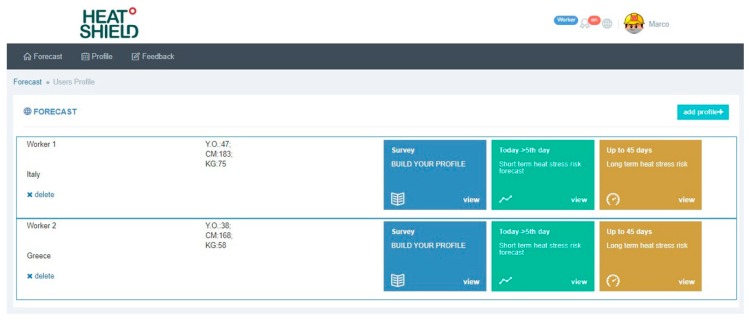
Page showing different worker’s profiles.

By accessing the “Profile” page, the user can also create multiple profiles for workers with different characteristics or engaged working in different geographical areas and therefore with different forecasts (Figure 8). 

Finally, by accessing the “Feedback” page, the user can also send feedback messages on various topics concerning the HEAT-SHIELD platform and report any errors.

### 3.2. WBGT Forecast Verification

**Figure 9:**
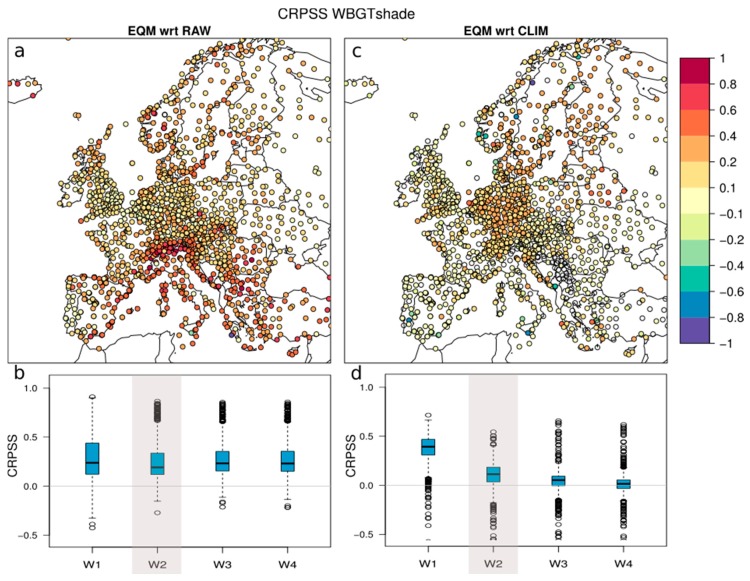
(**a**,**c**) Maps of skill score (SS) relative to a reference forecast (CRPSS) of WBGT_shade_ at lead times 12–18 days (Week 2 (W2), see details in Section 2.3.3), with respect to the raw forecasts (empirical quantile mapping (EQM) with respect to RAW) and to the climatology forecast (EQM with respect to CLIM). (**b**,**d**) Weekly CRPSS of WBGT_shade_. Each box represents the CRPSS values across European stations. The box for W2 (12–18 days) corresponds to the values displayed in the maps above.

The CRPSS is used to account for the skill of the forecasts used by the HEAT-SHIELD platform (Section 2.3.3). Figure 9a,c shows the spatial distribution of the CRPSS of WBGT_shade_ in week 2 (days 12 to 18). There is a clear added value of the bias-corrected forecasts with respect to the raw counterparts (positive CRPSS in Figure 9a). This added value appears mainly in regions with complex topography or at some coastal regions (Figure 9a) and remains at all lead times (Figure 9b), illustrating the capability of EQM to downscale the coarse model output to location-specific information. The bias-corrected forecasts are more skillful than climatology up to 12–18 days at most sites (Figure 9d), especially in Central and Northern Europe (Figure 9c). The CRPSS of WBGT_sun_ vs. climatology is very similar to that of WBGT_shade_ (not shown), both in terms of spatial pattern and skill decrease with lead time. For 19–25 days ahead, forecasts can only marginally add value to climatological forecasts, and beyond 25 days, forecasts are as skillful as climatology (Figure 9d). Note that a location-specific climatological forecast of WBGT also represents valuable information (i.e., location- and season-specific climatological risk of heat stress) and the HEAT-SHIELD platform therefore provides meaningful information throughout the full forecast range.

## 4. Discussion

The HEAT-SHIELD platform [24] developed within the frame of the European Project HEAT-SHIELD and described in this study represents the first step to fill the lack of international heat warning systems specifically addressed to occupational sectors. This website platform was officially launched in 2018 and is currently operating for about 1800 European localities. It represents the first international example of personalized short- and long-term heat risk forecasts with useful heat-related adaptation information for workers and stakeholders in charge of safeguarding workers’ health and productivity. 

The main characteristics of the HEAT-SHIELD platform are listed below and make this heat warning system original and unique:

- the HEAT-SHIELD platform is multilingual.
- The local-heat-stress-risk forecast is “customized” based on:
  ○ the worker’s physical characteristics (specifically height and weight),
  ○ the physical activity level,
  ○ the clothing or PPE worn during work,
  ○ the work environment (outdoors in the sun or shade),
  ○ also taking into account whether the worker is acclimatized or not to the heat.
- The short-term heat risk forecast (5-day forecasts) includes behavioral recommendations related to how much hydration (water intake) and rest (work breaks) during the worst (in term of heat stress) hour of the day.
- Long-term heat risk forecasts are available up to just over one month (46 days).

Currently the website platform is available in six languages (English, Italian, Slovenian, French, Portuguese and German) and will be further implemented in other languages. This characteristic is of great importance especially in the occupational field because most European countries are typically multicultural. It is indeed known that foreign workers may have a real difficulty in understanding the local language with consequent important repercussions on the perception of the heat risk in the workplace [64,65]. As reported in a recent review on the existing HHWSs in Europe [17], one of the main communication limits of these systems is that the warnings are generally issued in the local language of each country in addition to (in very few countries) English.

The non-customized heat stress forecast output (the maps of the weekly maximum probability of exceeding the daily WBGT_sun_ threshold of 27 °C) is simplified, generalized information valid for the whole Europe and accessible to everyone without any registration information. However, this information has the limitation of highlighting the potential heat risk mainly in southern Europe, displaying a clear latitudinal gradient typically resembling the air temperature gradient. The scientific literature [66,67,68] has shown that local populations are acclimatized to their local climate and respond to heat stress differently. A solution to try to solve this limitation would be to collect data on the perception of heat stress in the occupational sectors in various geographical areas with different climatic characteristics. In this way, the WBGT thresholds might be recalibrated accounting for geographical adaptation. The main aim of the very general information provided by the non-customized outputs is to motivate the user to register on the HEAT-SHIELD platform to obtain personalized information on heat stress risk calculated by using a tailored WBGT threshold based on individual worker characteristics and the workplace environment. The personalization of the forecast certainly represents an ambitious challenge to improve the generic information already available and provided by the main meteorological services and that need to correctly interpret each personal situation. This customized approach is essential in occupational settings due to the high variability of environmental conditions and job/task activities, which results in a strong heterogeneity of the thermal stress exposure with direct repercussions on workers’ health and productivity. However, it must also be considered that, in common practice, workers accustomed to carry out specific work tasks repetitively, might make a self-evaluation in a way that underestimates the work effort and therefore the heat-related job risk perception. This situation might include some bias and distort the efficiency of the strategies to counteract heat stress. Distortion might also depend on socio-cultural aspects, such as the dietary habits that underlie the maintenance of a good level of hydration and nutrition. For example, people of Muslim origin are at greater heat-related health risk during the Ramadan period [69,70]. On the other hand, a natural reaction to heat of a worker is to reduce their physical activity, that is a self-pacing or autonomous adaptation which reduces the body’s internal heat production but also the hourly work capacity [71,72,73].

A strength of the developed website platform relates to its ability to provide hydration and work/break schedule recommendations in the short term. Taking breaks in shady or cool areas as well as suggestions on hydration (water consumption) during working time according to specific heat stress conditions and physical efforts represents a fundamental heat-related adaptation method recommended by the ISO [36] and other governmental agencies [19,58]. Moreover, the platform also includes e-mail alerts that represent important adaptation strategies to timely counteract heat stress conditions and safeguard the workers’ health and productivity. 

The HEAT-SHIELD platform is a potentially very useful tool because heat stress is significantly increasing in many geographical areas worldwide, with strong effects also in European cities [74]. In addition, heat stress is expected to increase significantly in the next years because of climate change [1,2] also in areas where the worker population is not used to fighting this phenomenon [75], such as central-northern European countries.

At the moment, the HEAT-SHIELD platform is the only example of a website platform providing such a comprehensive collection of information. Nevertheless, there are already a few interesting smartphone applications in place that inform workers about precautions against outdoor heat stress. These include the OSHA NIOSH Heat Safety Tool [76] and the ClimApp [77] device currently in an advanced stage of development by several HEAT-SHIELD partners. These applications are, however, not able to provide long-term forecasting information that is particularly useful for planning issues. Precisely for this reason, the HEAT-SHIELD website platform is based on the extended range ensemble forecasts of the ECMWF that enables customized heat stress risk up to over a month. In this way, useful information for employers, organizations and operators in charge of safeguarding health and productivity in various occupational areas are provided, calibrated with greater precision, the interventions to be taken according to the subjective characteristics of the worker and other situations in which the workers are involved. A further interesting feature of the HEAT-SHIELD platform is the possibility for real-time verification of the heat stress risk situation by modifying some characteristics, for example, by varying the work environment (e.g., working in the shade) or the clothing worn, in this way planning the best actions to counteract the effects of the heat in the long term. Certainly, there is a need of further validation including the worker’s health component linked to the information provided by the HEAT-SHIELD platform. This might be done by processing the subjective information collected by the self-administered questionnaire developed within the frame of the HEAT-SHIELD project. They have already been used in several European countries for gathering evidences on workers’ risk perception of heat stress in the workplace and potential productivity losses due to extreme heat. For example, based on a preliminary investigation [78] carried out during the summer months of 2017 and 2018 on some workers engaged in Italy in construction and agriculture sectors, results revealed agreements between the ISO-standard WBGT thresholds associated with specific work efforts and the worker’s thermal stress perceptions for high WBGT values (WBGT > 30 °C). Conversely no agreements were observed for lower WBGT values. In the latter case, workers declared a heat stress level (from low to more often moderate heat stress) even if the ISO-standard WBGT threshold for that activity level does not recommend critical heat-stress conditions. For this reason, if data collected also in other countries during the summer of 2019 and the following summers confirm these preliminary results, the recalibration of the ISO-standard WBGT thresholds may be desirable, also including lower critical values which, however, may represent a health problem. Furthermore, through the case studies planned as part of the HEAT-SHIELD project, other health/physiological data of workers are being collected that could be of great help for a worker’s health validation in relation to heat stress in workplaces.

Since the HEAT-SHIELD project started (January 2016), some stakeholder meetings presenting the HEAT-SHIELD platform have already been organized during the years of 2018 and 2019 in several European countries, and other meetings are scheduled in different countries by the end of the project (December 2020). One of the main objectives of these meetings, in which employers, workers, worker safety representatives, prevention, protection service managers and competent doctors took part, was to obtain immediate feedback on the HEAT-SHIELD platform. Initial user feedback suggest that there is potential for further improvements, with new procedures/suggestions aimed to provide increasingly detailed information useful for worker’s heat-related health prevention and reducing productivity loss. Trying to maximize employers’ involvement in the use of the HEAT-SHIELD platform is a priority since they are considered key elements among all stakeholders. In particular, employers are the main actors for regulating work activities (i.e., defining of the length of work shifts and relative work breaks, identifying of the days and working hours in which to carry out certain work activities, defining of the number of workers involved in specific work tasks, etc.) and are responsible for the workers’ health, without ever losing sight of the economic aspect linked to work productivity. 

Field studies carried out also in the field of the HEAT-SHIELD project [14,20,22,65,79,80,81] aimed at evaluating the responses of workers exposed to heat stress conditions during different work activities will be particularly useful for identifying the best heat-related adaptation strategies helpful to manage this hazard situation. For now, only recommendations on water consumption and work/rest breaks clearly described in reports provided by international organizations working on this topic are provided. However, other recommendations (i.e., the recommended clothing, or others) obtained by using other thermal-stress indicators, subjective information (i.e., age or gender), and detailed infographics related to specific occupational sectors, could also be integrated and included in an operational way in the HEAT-SHIELD platform to counteract the effects of heat. A more complex issue, on the other hand, concerns the possibility of personalizing the heat risk level based on pre-existing diseases or specific pharmacological treatments. Currently, information on the customized heat risk refers to a healthy worker and not where specific drugs are used; the situation should always and exclusively be evaluated by an occupational health physician. 

In the near future, it will also be desirable to develop a system for monitoring work injuries to be updated in real time, to report heat-related injuries in order to activate timely emergency response interventions. In the current version, the website platform does not include heat stress thresholds based on the relationships between WBGT and injuries because only very few studies have investigated this relationship [82]. In addition, the use of meteorological data for occupational heat stress assessment is actually limited because weather stations do not traditionally and directly measure some important climate factors useful for WBGT calculation [83,84]. For this reason, results are not as obvious as those identified between several thermal indicators and some categories of the general population (e.g., the elderly) [85,86,87]. In fact, in this latter case, city-specific thermal stress thresholds were identified, and in several cases, these thresholds were implemented in HHWSs addressed to the general population or the elderly [17]. 

The current website platform relies on a probabilistic forecast model, which has the advantage of allowing long-term forecasts. It has, however, also some limitations such as the temporal resolution (it only provides a daily value). In particular, the intra-daily hourly heat stress risk forecast (i.e., morning, afternoon, evening, night) is not provided and the information is only available for a limited number of European localities (about 1800). For this reason, we are already working to implement the heat stress risk forecast in the short term for specific regions by using high-resolution (i.e., spatial resolution of 3–7 km) deterministic meteorological models. In this way, detailed information on an hourly basis will be obtained and personalized heat stress risk will be available for various times of the day in which workers can be engaged. Furthermore, by exploiting the high spatial resolution of deterministic meteorological models, the information will be extended to all locations without the need to perform downscaling operations at the meteorological station level.

## 5. Conclusions

The HEAT-SHIELD platform [24] is the customized occupational heat-related warning system developed within the framework of the European HEAT-SHIELD project as the first operational website platform providing short- and long-term heat warning to safeguard workers’ health and productivity on a continental scale. This platform represents a useful adaptation strategy aimed at protecting workers, a population category particularly exposed to the effects of climate change. The usefulness of this type of adaptation strategies is linked to the fact that, based on future climate change scenarios, more and more workers operating on ever-wider geographical areas affected by heat-stress hazard conditions will be exposed for longer periods of time during the year to the effects of global warming.

## Figures and Tables

**Table 1:** The characteristics of the HEAT-SHIELD platform risk levels.

HEAT-SHIELD Platform Risk Levels and Color Codes	WBGT Levels		Work Breaks	Water Consumption (Hydration)	HEAT-SHIELD Recommendations
	Unacclimatized	Acclimatized			
Not significant RL ≤ 80%	<22.5 L_MR_ <20.0 M_MR_ <18.5 H_MR_ <17.5 VH_MR_	<25.0 L_MR_ <23.0 M_MR_ <21.5 H_MR_ <20.5 VH_MR_	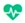	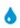 L_MR_ 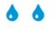 from M_MR_ to VH_MR_	No special precautions are required: Maintain normal working and hydration procedures.
Low 80% < RL < 100%	22.5 L_MR_ 20.0 M_MR_ 18.5 H_MR_ 17.5 VH_MR_	25.0 L_MR_ 23.0 M_MR_ 21.5 H_MR_ 20.5 VH_MR_	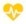	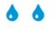 L_MR_ and M_MR_ 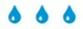 H_MR_ and VH_MR_	Pre-alarm (attention): Pay attention to frequent drinking and plan small breaks.
Moderate 100% ≤ RL < 120%	28.5 L_MR_ 25.0 M_MR_ 23.0 H_MR_ 22.0 VH_MR_	31.0 L_MR_ 28.5 M_MR_ 27.0 H_MR_ 25.5 VH_MR_	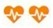	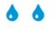 L_MR_ and M_MR_ 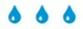 H_MR_ and VH_MR_	Alarm: Drink frequently and increase the number of breaks with cooling.
High RL ≥ 120%	>33.5 L_MR_ >29.5 M_MR_ >27.5 H_MR_ >25.5 VH_MR_	>36.5 L_MR_ >33.5 M_MR_ >31.5 H_MR_ >30.5 VH_MR_	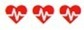	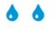 L_MR_ and M_MR_ 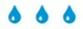 H_MR_ and VH_MR_	Emergency: Drink often, even more than 1 L/h and schedule frequent breaks in shadowed or cool area.

L_MR_, M_MR_, H_MR_ and VH_MR_ represent low (180 W), moderate (300 W), high (415 W), and very high (520 W) metabolic rates (MR), respectively. Green heart: No further breaks than usual are required; Yellow heart: Plan small breaks; Two orange hearts: Increase the number of breaks; Three red hearts: Frequent breaks. One drop: Drink about half a liter of water per hour; Two drops: Drink about a liter per hour; Three drops: Drink more than a liter of water per hour.
